# 3D-Quantification using an interleaved Look-Locker acquisition sequence with T2-prep pulse (3D-QALAS)

**DOI:** 10.1186/1532-429X-16-S1-O82

**Published:** 2014-01-16

**Authors:** Sofia Kvernby, Marcel Warntjes, Carl-Johan Carlhäll, Jan Engvall, Tino Ebbers

**Affiliations:** 1 Linköping University, Linköping, Sweden; 2 Center for Medical Image Science and Visualization (CMIV), Linköping, Sweden

## Background

Quantification of the longitudinal- and transverse relaxation time in the myocardium has shown to provide important information in cardiac diagnostics. Several methods are currently available for cardiac relaxation time mapping but they generally demand a long breath hold to measure either T1 or T2 in a single 2D slice. In order to make quantification of relaxation parameters in the myocardium clinically applicable, the methods must be improved in speed and coverage. In this work we propose a novel method for 3D interleaved T1 and T2 quantification of the whole left ventricular myocardium within one single breath hold.

## Methods

The sequence is based on a 3D spoiled Turbo Field Echo sequence using inversion recovery with interleaved T2 preparation and is referred to as 3D-QALAS (3D-quantification using an interleaved Look-Locker acquisition sequence with T2 preparation pulse). Quantification of both T1 and T2 in a volume of 13 slices with a resolution of 2.0 × 2.0 × 6.0 mm is obtained. The acquisition time is restricted to 230 ms during end diastole to avoid artifacts from cardiac motion. Data acquisition is performed once after a T2 preparation pulse and four times after a T1 sensitizing inversion pulse (5 heartbeats). This acquisition scheme is repeated three times to sample the 3D k-space, resulting in a breath hold of 15 heartbeats. The sequence was validated in both phantoms and healthy volunteers using a Philips Ingenia 3T scanner. The effect of different heart rates, flip angles and deliberately applied arrhythmias were simulated and investigated in phantoms.

## Results

**Figure 1:**
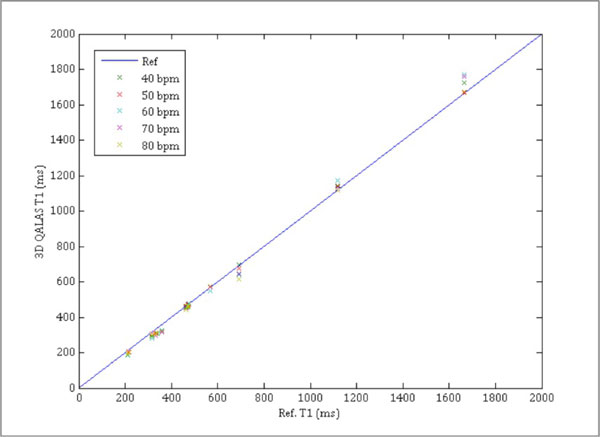
**Measurements of T1 in phantoms with 3D QALAS and the corresponding reference T1 for specific simulated heart rates**.

**Figure 2:**
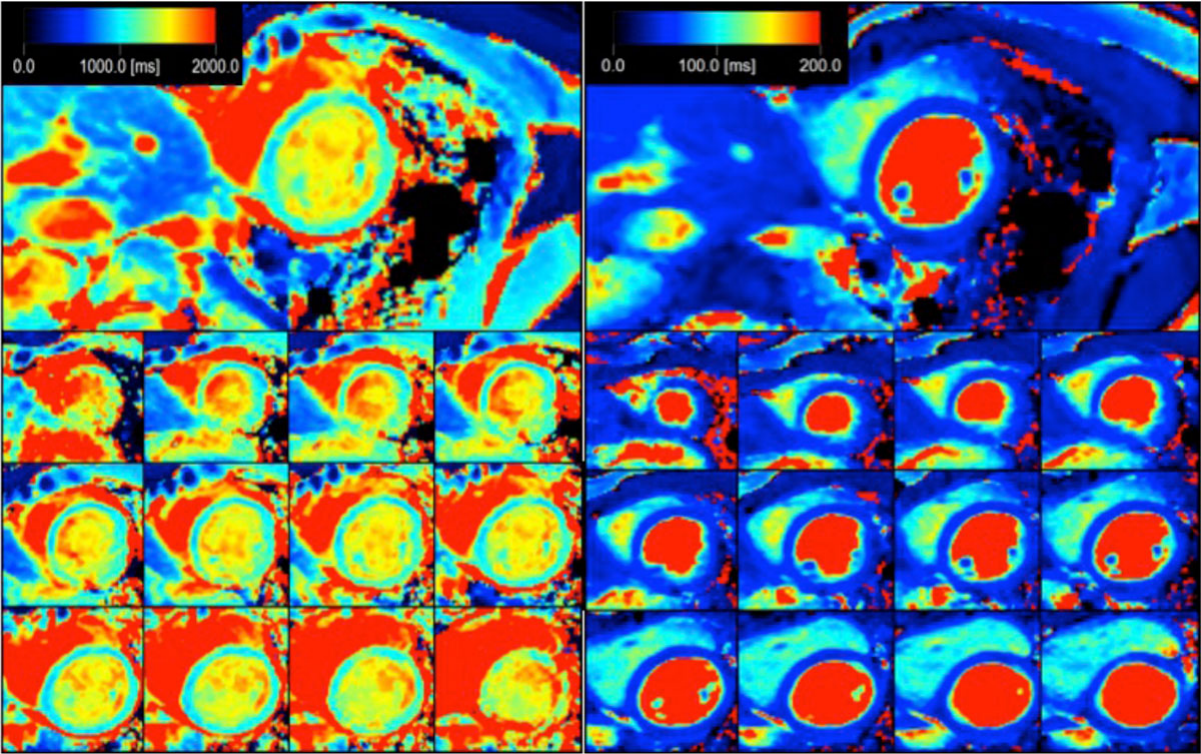
**3D QALAS T1 maps (left) and T2 maps (right) of 13 slices for a healthy volunteer**.

Relaxation times in phantoms correlate well with reference methods, Inversion Recovery for T1(R = 0,999) and Multi Echo for T2(R = 0,960). Quantification of T1 and T2 using 3D QALAS showed no dependence on heart rate, flip angle or arrhythmia within the clinically relevant range. In vitro T1 measurements with 3D QALAS for different heart rates, which has shown to be challenging for other methods, are shown in Figure 1. In Figure 2, T1- and T2 maps are shown for a healthy volunteer. The mean native myocardial T1 and T2 values in this volunteer were 1085 ± 39 ms (mean ± SD) and 52 ± 5 ms which is in accordance with previous results (Piechnik S. et Al, 2010, JCMR; Shivraman G. et Al, 2009, JCMR).

## Conclusions

3D QALAS allows quantification of both T1 and T2 in the whole left ventricular myocardium within one breath hold, making T1 and T2 quantification clinically applicable to a broader spectrum of diseases.

## Funding

Swedish Research Council and Swedish Heart-Lung Foundation.

